# Identification of Cadmium Contamination in Rice Using Near-Infrared Reflectance Spectroscopy and Machine Learning

**DOI:** 10.3390/foods15173000

**Published:** 2026-08-26

**Authors:** Xuexue Miao, Ying Miao, Ni Li, Yang Liu, Weiping Wang

**Affiliations:** 1 State Key Laboratory of Hybrid Rice, Hunan Hybrid Rice Research Center, Changsha 410125, China; 2 Yuelushan Laboratory, Changsha 410125, China; 3 School of Computer Science & Technology, Beijing Institute of Technology, Beijing 100081, China; 4 Longping College of Agriculture, Hunan University, Changsha 410082, China

**Keywords:** near-infrared spectroscopy, rice, cadmium, variable selection, food safety

## Abstract

Routine monitoring of cadmium (Cd) contamination in rice is essential for public health protection and agricultural trade security. Conventional chemical detection methods are environmentally unfriendly, labor-intensive, and slow. This study presents a rapid, accurate classification approach based on near-infrared reflectance spectroscopy (NIRS) for discriminating Cd-contaminated rice from uncontaminated rice. Five spectral preprocessing methods and three variable selection algorithms were systematically evaluated for their influence on model performance. Classification models were developed using partial least squares discriminant analysis (PLS-DA), K-nearest neighbors (KNN), and support vector machines (SVM). Second derivative (2D) preprocessing yielded the greatest performance gains, raising KNN and SVM test-set accuracy from 73% and 88% to 93% and 91%, respectively. Among the variable selection strategies, the successive projections algorithm (SPA) proved most effective. Under optimized conditions, PLS-DA achieved the best overall performance, attaining 92% accuracy, 89% specificity, and 95% sensitivity on the test set. These results demonstrate the strong potential of NIRS coupled with machine learning for rapid, large-scale Cd surveillance in rice, providing robust technical support for grain quality monitoring and low-cadmium variety breeding programs.

## 1. Introduction

Rice (*Oryza sativa* L.) is the primary caloric staple for more than half the world’s population, particularly across Asia [1], making its yield and quality central to global food security and economic stability. Over recent decades, Cd contamination of rice has escalated sharply owing to the expansion of mining activity and the widespread use of Cd-laden wastewater for irrigation [2,3]. As a persistent, non-biodegradable toxic metal, Cd bioaccumulates through the food chain and poses severe health risks to consumers. Cadmium has a long-lasting biological half-life of 16 to 30 years inside human bodies. Slow, low-dose cadmium poisoning may cause hypertension and chronic respiratory illnesses. Its long-term exposure induces cancers, diverse central nervous system disorders, bone-related ailments, and teratogenic impacts [4]. The historical “itai-itai” epidemic in Japan, attributed to chronic ingestion of Cd-contaminated rice from the 1910s through the 1970s, remains a defining illustration of these hazards [5]. In response, regulatory limits have been established worldwide: China permits a maximum Cd concentration of 0.2 mg/kg in rice, while the European Union enforces a stricter ceiling of 0.1 mg/kg [6]. Given the direct threat Cd poses to food safety and human health, robust routine surveillance of rice Cd levels is indispensable.

Cd-contaminated rice is visually indistinguishable from clean grain, precluding any naked-eye screening. Current monitoring relies predominantly on destructive chemical techniques, including atomic absorption spectrophotometry (AAS) [7], inductively coupled plasma optical emission spectrometry (ICP-OES) [8], and inductively coupled plasma mass spectrometry (ICP-MS) [9]. These methods demand laborious sample digestion and multi-step pretreatment, rendering them time-consuming, costly, and environmentally burdensome. Advances in molecular biology have introduced DNA-based alternatives such as Kompetitive Allele-Specific Polymerase Chain Reaction (KASP-PCR) genotyping for identifying low-Cd-accumulating rice varieties; KASP markers targeting OsNRAMP5, the primary gene encoding a Cd transporter in rice, have been successfully developed [10,11]. However, molecular marker assays are typically performed at the seedling stage, and their correlation with actual Cd concentrations in mature grain requires further validation. None of these approaches supports the rapid, real-time, large-scale screening that modern supply chains demand, underscoring the need for a simple, high-throughput alternative.

Near-infrared reflectance spectroscopy (NIRS) is a well-established rapid analytical technology that circumvents the drawbacks of conventional methods by enabling non-destructive, reagent-free measurement. NIR spectra encode rich information on organic molecular structure through combination and overtone absorption bands of O–H, C–H, C–O, and N–H bonds. Integrated with chemometric tools, NIRS has been broadly applied across food, agriculture, and environmental domains [12,13,14]. In rice quality assessment specifically, NIRS has successfully discriminated grain quality grades [15,16], storage duration [17,18], and variety [19,20]. Although heavy metals lack NIR absorption bands, they form complexes with proteins and other biomolecules in plant tissue, generating indirect spectral signatures detectable in the NIR region [21]. Several studies have exploited this principle: Liu et al. achieved mean classification accuracies exceeding 95.67% for NIRS-based discrimination of mussels contaminated with Zn, Pb, Cd, and Cu using extreme learning machines [22]; Huang et al. quantified six heavy metals including Cd in dark sun-cured tobacco by NIRS, and the determination coefficient of prediction (R_p_^2^) and root-mean-square error of prediction (RMSEP) for Cd reached 89.10% and 2.051 mg/kg, respectively [23]; Wang et al. (2024) and Gao et al. (2023) demonstrated NIRS as a rapid, low-cost screening tool for Cd in peanut oil and kernels [24,25], respectively. Despite this evidence, the literature specifically addressing NIRS-based Cd detection in rice remains scarce.

A range of chemometric methods has been applied to NIR-based qualitative classification, including principal component analysis (PCA) [26], partial least-squares discriminant analysis (PLS-DA) [27], support vector machine (SVM) [28], and K-nearest neighbors (KNN) [29]. Nevertheless, the high dimensionality and collinearity inherent to full-spectrum NIR data introduce redundant variables that degrade model parsimony and predictive performance. Variable selection addresses this by isolating the most informative wavelengths; established algorithms include interval PLS, synergy interval PLS, backward interval PLS, competitive adaptive reweighted sampling (CARS) [30], and the successive projections algorithm (SPA) [31]. Complementing these approaches, two-dimensional correlation spectroscopy (2DCOS) enhances spectral resolution and resolves band overlap inherent in one-dimensional spectra [32], making 2DCOS a powerful tool for guiding targeted variable selection in NIR modeling.

In this study, NIRS combined with machine learning was explored for binary classification of Cd-contaminated versus uncontaminated rice. We first characterized spectral differences between sample classes, then evaluated five preprocessing methods and three variable selection strategies (CARS, UVE, and SPA) to optimize spectral input. We then constructed classification models using PLS-DA, SVM, and KNN, and assessed them by sensitivity, specificity, and accuracy. The specific objectives were to: (1) evaluate the capability of NIRS to discriminate Cd-contaminated rice; (2) identify Cd-associated characteristic wavelengths from the full spectrum using multiple variable selection strategies; (3) benchmark the performance of three machine learning classifiers; and (4) determine the optimal classification model. Beyond ensuring consumer safety, rapid identification of Cd-contaminated rice offers direct technical support for breeding low-cadmium varieties, with broad implications for food security and sustainable agricultural production.

## 2. Materials and Methods

### 2.1. Sample Preparation

A total of 238 rice samples were collected from six regions of Hunan Province, China, namely Yueyang (45), Changde (32), Huaihua (37), Zhuzhou (41), Yiyang (36), and Xiangtan (47), between September and November 2025. The rice cultivars included 7 varieties: Huanghuazhan, Nongxiang 42, Yuzhenxiang, Jingliangyou534, Longliangyouhuazhan, Taoyouxiangzhan, Taiyounong 39. Among these samples, 118 Cd-contaminated and 120 uncontaminated samples were included. Soil Cd concentrations across these areas ranged from 0.03 to 2.0 mg/kg, producing a corresponding gradient of Cd levels in the harvested grain. We collected each rice sample from a separate paddy field. All samples were oven-dried at 40 °C for 24 h to perform NIR spectral acquisition. Then, the samples were milled to a fine powder using a CT410 mill (FOSS, Hillerød, Denmark) for reference chemical analysis.

### 2.2. Reference Measurement

For each sample, 0.5 g of rice powder was weighed into a PTFE digestion vessel along with 6 mL of nitric acid. Microwave digestion was performed using a MARS 6 system (CEM, Matthews, NC, USA) with a temperature ramp to 190 °C over 20 min, followed by a twenty-minute hold. After cooling, digests were diluted to 50 mL with ultrapure water and Cd concentrations were quantified by ICP-MS (iCAP RQ, Thermo Fisher, Waltham, MA, USA). Calibration was performed with five standard concentrations (0.5, 1.0, 5.0, 10.0 and 50.0 μL/L), producing a coefficient of determination (R^2^) of 0.9999 for the linear fit. Spike recoveries ranged from 96.5% to 99.3%, and relative standard deviations (RSDs) ranged from 1.2% to 2.1% (Table S1). Precision validation via certified reference material GBW10010 gave an RSD of 0.77% over six replicate measurements. The ICP-MS limit of detection (LOD) and limit of quantification (LOQ) were 0.00145 mg/kg and 0.0048 mg/kg, respectively. Moreover, the threshold for contaminated and non-contaminated samples was 0.2 mg/kg. According to this criterion, samples with cadmium concentrations > 0.2 mg/kg are classified as contaminated, and those ≤0.2 mg/kg as uncontaminated.

### 2.3. Spectral Collection

**Figure 1:**
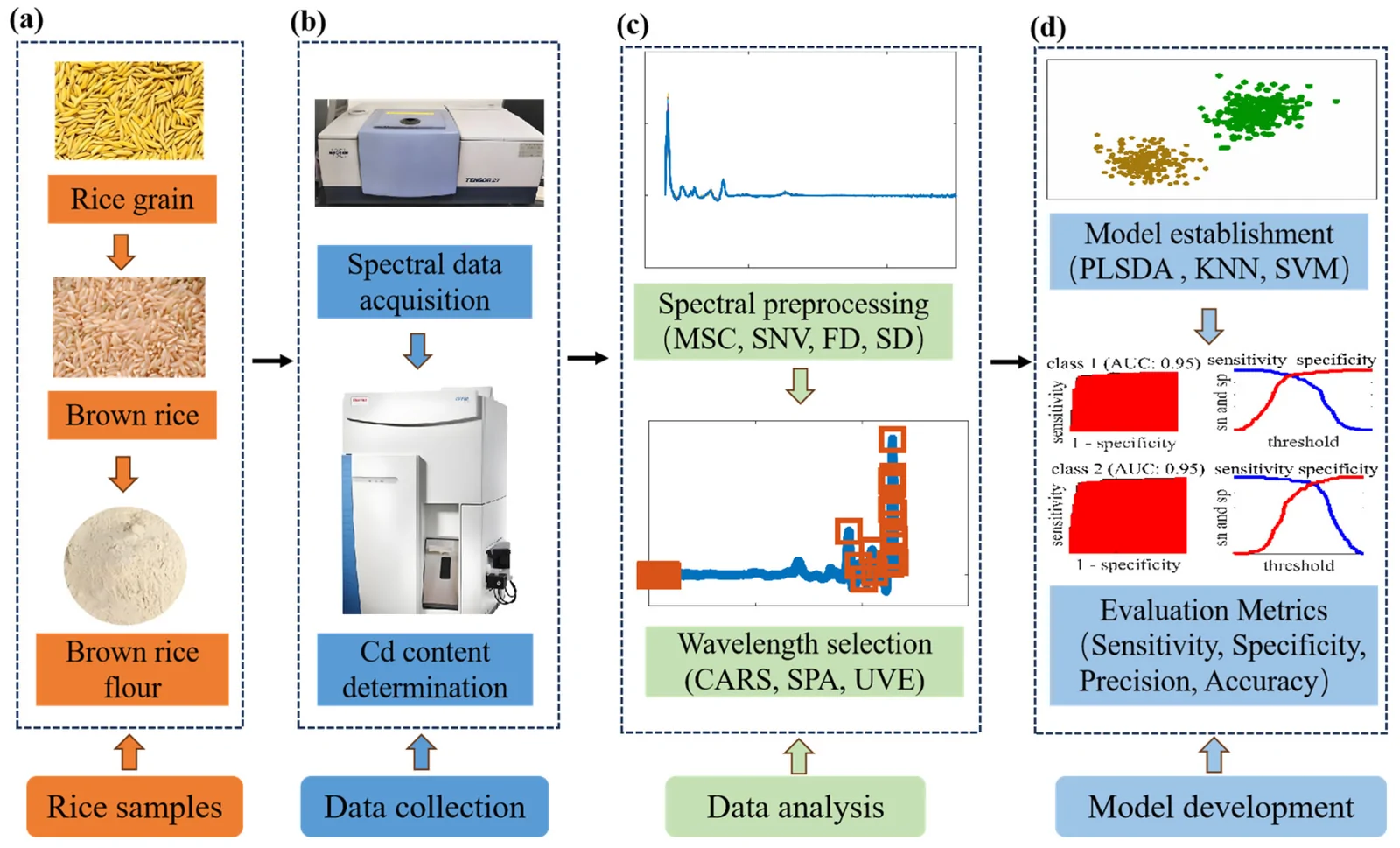
Process of constructing NIR classification models. (**a**) Preparation of rice samples; (**b**) acquisition of spectral and chemical data; (**c**) optimization of the model; (**d**) model construction and evaluation.

NIR spectra were acquired using a Fourier-transform NIR spectrometer (Matrix-I, Bruker Optics, Ettlingen, Germany) operated with OPUS 6.5 software. For each measurement, 30 g of rice powder was packed into a circular sample cup and spectra were collected in diffuse reflectance mode via an integrating sphere. A background reference scan was recorded prior to each session to minimize environmental interference. Spectra were collected over the range 4000–12,000 cm^−1^, yielding 1154 spectral variables, with each spectrum representing the co-addition of 64 scans at a resolution of 8 cm^−1^. All measurements were performed in triplicate, and the mean spectrum was used for subsequent analysis. Figure 1 illustrates the overall workflow for constructing NIR classification models.

### 2.4. Spectral Preprocessing

Environmental stray light and instrument instability introduce random noise and baseline drift into raw NIR spectra. Appropriate preprocessing is therefore essential to correct baseline offsets and resolve overlapping absorption bands. Five preprocessing methods were evaluated: first derivative (FD), second derivative (SD), multiplicative scatter correction (MSC), standard normal variate (SNV), and normalization. FD and SD transformations suppress baseline drift and sharpen overlapping peaks, while MSC and SNV reduce particle-size-induced light scattering effects.

### 2.5. Variable Selection

Adjacent wavelengths in NIR spectra are highly collinear, creating substantial information redundancy that can impair classifier performance. Variable selection prior to model construction is therefore necessary to retain only the wavelengths most informative for Cd discrimination. Three selection strategies were evaluated: CARS, uninformative variable elimination (UVE), and SPA.

CARS iteratively identifies the most analytically relevant variables through a Monte Carlo sampling and regression-weight optimization strategy [33]. In each iteration, a random subset of the spectral data is drawn and a PLS regression model is fitted; variables contributing minimally to model performance are progressively eliminated based on their regression coefficients, and the variable subset yielding the lowest cross-validation error is retained as optimal [34].

SPA minimizes inter-variable collinearity by selecting a compact subset of spectrally distinct wavelengths. Starting from an initial variable, the algorithm iteratively selects the wavelength vector with the largest projection onto the subspace orthogonal to all previously selected vectors, ensuring each added variable contributes unique, non-redundant information. The optimal subset size is determined by comparing the root mean square error of cross-validation (RMSECV) across candidate subsets and selecting the minimum.

UVE eliminates variables whose information content is no greater than that of random noise. Stability values for each variable are computed over 500 Monte Carlo cross-validation iterations, in each of which a PLS model is built on a randomly drawn 80% subset of the samples. A threshold is then defined as the maximum absolute stability value observed among artificially appended noise variables; original spectral variables falling below this threshold are discarded as uninformative.

### 2.6. Chemometric Analysis

#### 2.6.1. PCA

Prior to supervised classification, principal component analysis (PCA) was applied as an unsupervised dimensionality-reduction step to explore the natural structure of the data and visualize class separation between Cd-contaminated and uncontaminated rice [35]. The most discriminative information was captured by a small number of principal components (PCs).

#### 2.6.2. KNN

KNN is a straightforward instance-based supervised classifier that assigns an unknown sample to the majority class among its K nearest neighbors in the training set, where proximity is measured by Euclidean or Manhattan distance. Its simplicity and interpretability make KNN a useful baseline for benchmarking more complex algorithms.

#### 2.6.3. SVM

SVM constructs optimal separating hyperplanes in a high-dimensional feature space by maximizing the margin between classes. Input data are mapped into this space via a kernel function, enabling efficient non-linear separation without explicit high-dimensional computation. A radial basis function (RBF) kernel was employed here, given its well-established capacity to capture both linear and non-linear relationships between spectral features and class labels.

#### 2.6.4. PLS-DA

PLS-DA is a supervised classifier that finds latent variables maximizing the covariance between the spectral predictor matrix (X) and a numerically encoded class matrix (Y). Class labels were encoded as integers: 1 for Cd-contaminated rice and 2 for uncontaminated rice. The PLS regression algorithm then extracts latent variables capturing the shared variance between X and Y, and samples are classified by comparing predicted response values against predefined thresholds. The optimal number of latent variables was determined by twenty-fold cross-validation.

### 2.7. Model Evaluation

Classifier performance was evaluated using sensitivity, specificity, precision, and accuracy, all derived from the confusion matrix entries (TP, TN, FP, FN), where higher values indicate superior performance. The defining equations are:

(1) $$Accuracy=\frac{TP+TN}{TP+TN+FP+FN}$$

(2) $$Specificity=\frac{TN}{TN+FP}$$

(3) $$Sensitivity=\frac{TP}{TP+FN}$$

(4) $$Precision=\frac{TP}{TP+FP}$$

where TP, TN, FP, and FN denote the numbers of true positives, true negatives, false positives, and false negatives, respectively [36]. In this study, positive samples refer to Cd-contaminated rice samples, while negative samples denote non-contaminated rice samples. Additionally, the area under the receiver operating characteristic curve (AUC) was used to assess the discriminative capacity of the PLS-DA model, with higher AUC values indicating stronger class separation. Data analysis was performed using MATLAB software (version R2020 b, The MathWorks, Natick, MA, USA).

## 3. Results and Discussion

### 3.1. Cadmium Content in Rice Samples

According to the Chinese National Standard GB 2762-2022 [37], the maximum permissible concentration of Cd in rice is 0.2 mg/kg. Based on this threshold, the rice samples in this study were categorized into two groups: samples with Cd concentrations ≤ 0.2 mg/kg were classified as non-contaminated, whereas those with Cd concentrations > 0.2 mg/kg were defined as Cd-contaminated.

Table 1 presents the distribution and descriptive statistics of Cd concentrations in rice. Prior to model development, the 238 rice samples were divided into training and test sets at a 2:1 ratio using the Kennard–Stone algorithm [38]. The training set included 159 samples: 81 non-contaminated and 78 Cd-contaminated. Cd concentrations in the training set ranged from 0.005 to 1.86 mg/kg, with an average value of 0.39 mg/kg. The test set consisted of 79 samples, of which 37 were non-contaminated and 42 were Cd-contaminated. Cd concentrations in the test set ranged from 0.003 (<LOQ) to 1.83 mg/kg, with a mean value of 0.41 mg/kg.

The training set was used for model construction, whereas the test set served to evaluate model performance. Notably, the distribution of contaminated and non-contaminated samples remained balanced across both datasets, ensuring reliable model evaluation [39].

### 3.2. Spectral Characteristics of Rice Samples

NIR absorption bands primarily arise from hydrogen-containing functional groups associated with major rice constituents, including proteins, starch, and lipids. Therefore, in-depth spectral analysis provides a basis for constructing robust classification models.

**Figure 2:**
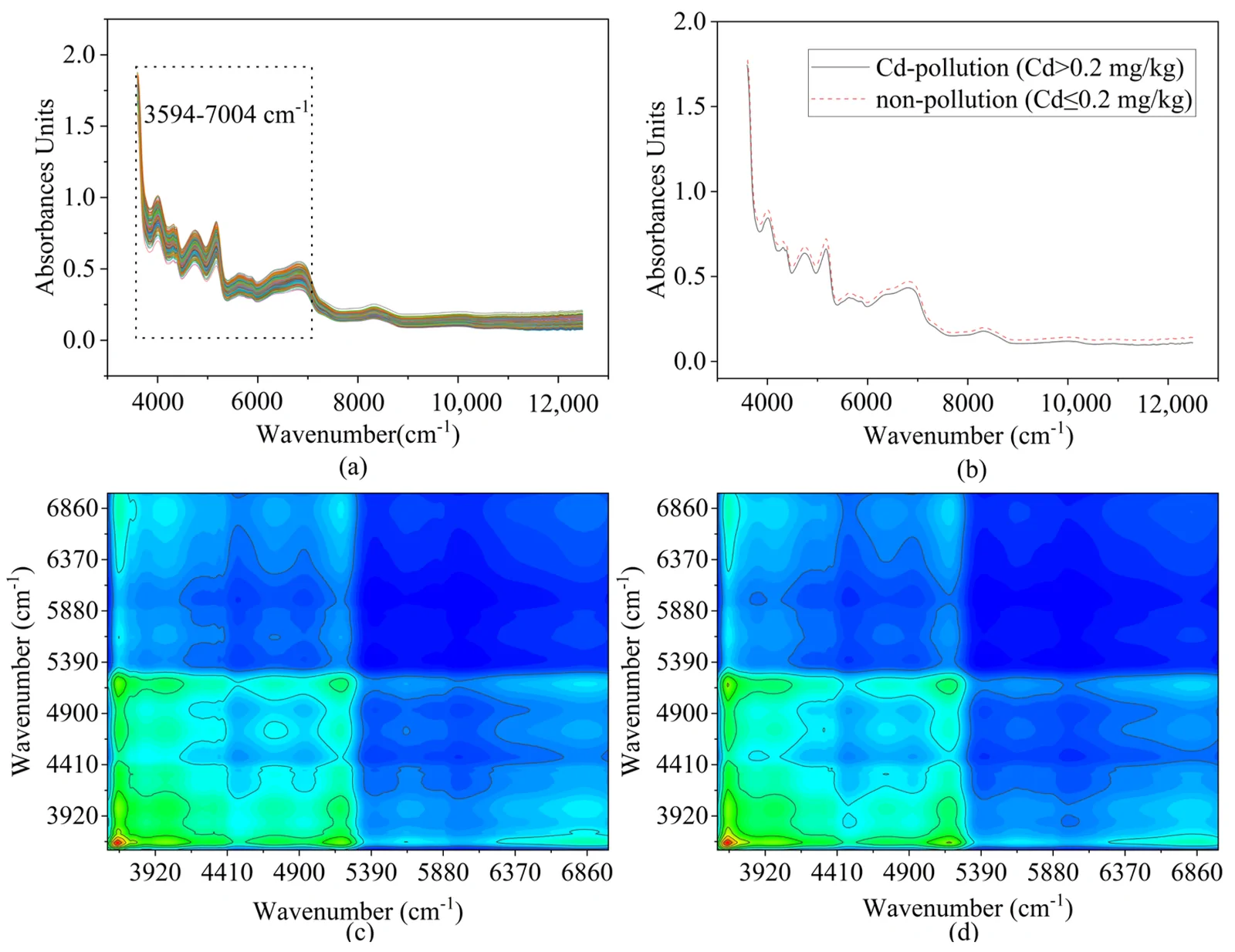
Original spectra of rice samples (**a**) and raw mean spectral maps of the Cd-contaminated and non-contaminated samples (**b**); Synchronous 2DCOS analysis flowchart of non-contaminated samples (**c**) and Cd-contaminated samples (**d**).

The raw NIR spectra of the 238 rice samples are shown in Figure 2a, where the overall spectral profiles exhibit similar shapes. Figure 2b presents the average spectra of Cd-contaminated and non-contaminated samples, revealing discernible differences in spectral characteristics between the two groups. Strong absorption features are observed within the 3594–7004 cm^−1^ region. Specifically, the 4920–4675 cm^−1^ region is associated with N–H stretching vibrations of proteins and O–H deformation in sucrose, while the 4400–3800 cm^−1^ region likely corresponds to the second overtone of C–H deformation vibrations in lipids.

Distinct absorption features are also observed near 4312 cm^−1^, 4720 cm^−1^, and 5194 cm^−1^, along with broader bands around 5555 cm^−1^, 5600 cm^−1^, 6849 cm^−1^, and 8300 cm^−1^. The peak near 5600 cm^−1^ is likely related to N–H and C–H stretching vibrations in proteins, whereas the absorption band around 8300 cm^−1^ corresponds to C–H stretching vibrations in lipids, starch, and proteins [40]. The signal at 6849 cm^−1^ is attributed to O–H bending vibrations associated with water, while the peak near 5555 cm^−1^ represents the first overtone of C–H stretching and CH_2_ groups [41].

The spectral region highlighted by the black box in Figure 2a exhibits substantial overlap among samples, making detailed interpretation difficult. To better resolve these features, we conducted 2DCOS analysis. The resulting 2DCOS maps for non-polluted and Cd-contaminated rice within the 3594–7004 cm^−1^ region are presented in Figure 2c,d. In synchronous spectra, positive correlations (red regions) indicate simultaneous changes in absorption intensity, whereas negative correlations (blue regions) represent opposite trends. Notable differences between the two classes appeared near 4700 cm^−1^ and 4300 cm^−1^, where distinct autopeaks emerged. These features may correspond to combined C–H stretching and CH_2_ deformation vibrations [42].

Overall, the spectral distinctions between contaminated and non-contaminated rice suggest that Cd contamination may alter the biochemical composition of rice, including proteins, starch, and lipids. These compositional changes are reflected in the NIR spectra, supporting the feasibility of distinguishing Cd-contaminated rice using spectroscopic techniques.

### 3.3. Exploratory Analysis Using PCA

**Figure 3:**
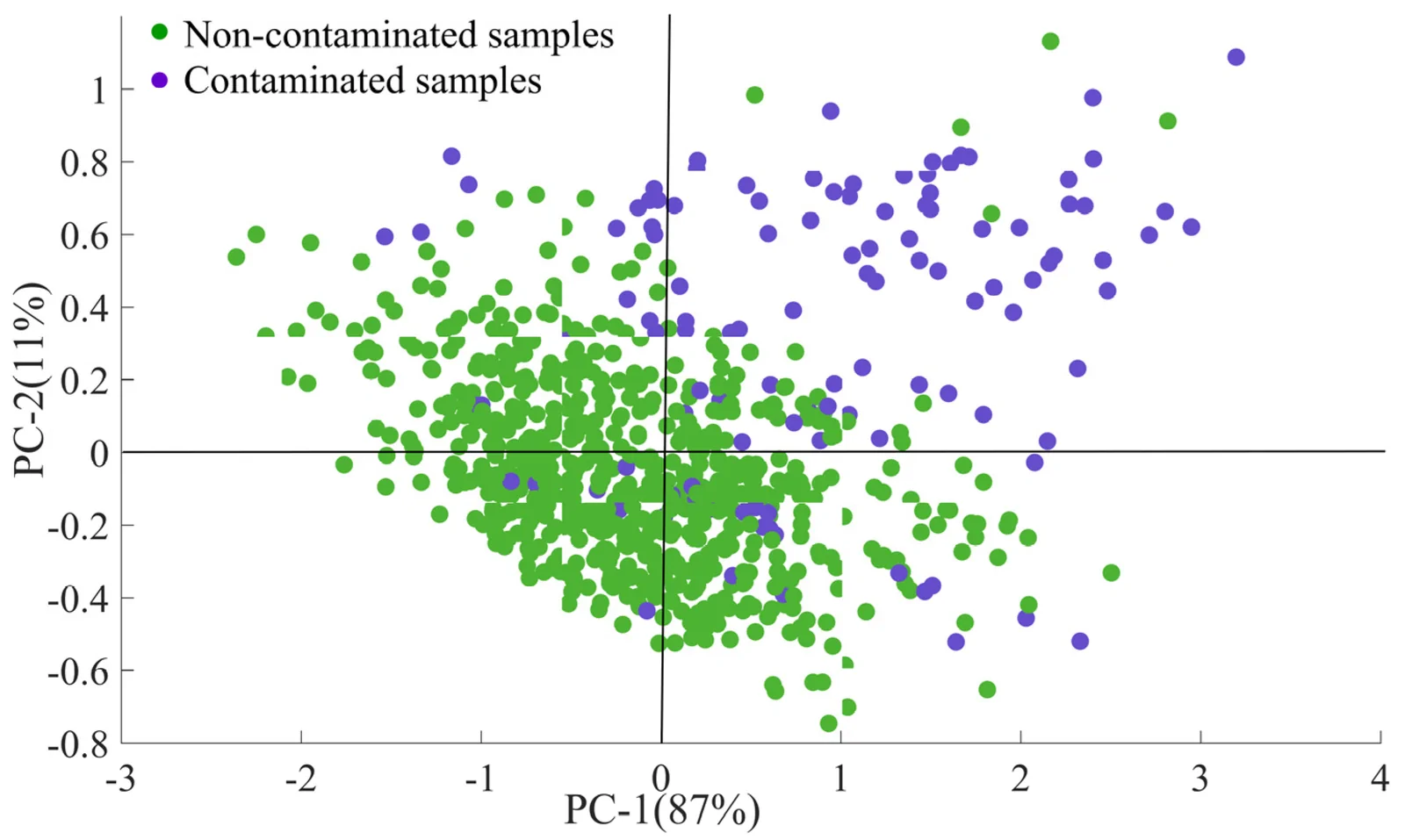
The scatter plot based on principal component analysis (PCA).

PCA was performed to explore potential clustering patterns among samples without prior variable selection. As shown in Figure 3, Cd-contaminated samples were primarily distributed in the positive region of the first principal component (PC1), whereas non-polluted samples tended to cluster in the negative region.

The first two principal components accounted for 98% of the total variance, indicating that PCA effectively captured the dominant spectral variation within the dataset. The scatter plot in Figure 3 reveals a clear tendency toward separation between Cd-contaminated and non-contaminated rice samples, suggesting that Cd contamination significantly influences the spectral characteristics of rice.

Despite this trend, partial overlap between the two groups remained, indicating that PCA alone is insufficient for complete discrimination. Consequently, supervised classification algorithms are required to achieve more reliable separation between polluted and non-polluted samples.

### 3.4. Classification Models Based on the Full Spectral Range

To evaluate the impact of spectral preprocessing on classification performance, KNN, SVM, and PLS-DA models were constructed using the full spectral range under various preprocessing conditions, without variable selection.

For the KNN algorithm, the K value was optimized within the range of 1–10, and the optimal value was determined by minimizing classification error. As shown in Table 2, results for the training and cross-validation sets were generally consistent with those for the test set. Using raw spectra, the classification accuracies for the training, cross-validation, and test sets were 76%, 78%, and 73%, respectively. After applying spectral preprocessing, model sensitivity, specificity, and accuracy improved substantially. Among the tested approaches, SD preprocessing performed best. Under this condition, the KNN model achieved 95%, 83%, and 89% sensitivity, specificity, and accuracy, respectively, for the training set, and 92%, 93%, and 93% for the test set. These results indicate that appropriate spectral preprocessing can markedly enhance the predictive capability of the KNN model.

For the PLS-DA model, the optimal number of latent variables (LVs) was selected based on the minimum classification error. Overall, the PLS-DA training models achieved satisfactory performance, with sensitivity, specificity, and accuracy generally exceeding 91%, except in a few cases (Table 2). However, model performance declined in the cross-validation and test sets, where accuracies ranged from 89 to 91% and 77–91%, respectively. Preprocessing methods such as FD, SD, MSC, and normalization did not substantially improve model performance. Although the FD-PLSDA and SD-PLSDA models achieved 100% accuracy in the training set, their accuracies dropped to 87% and 89%, respectively, in the test set, indicating potential overfitting. Among the evaluated methods, only the model processed by SNV has better stability, yielding 91% accuracy in both the training and test sets with 10 latent variables.

For the SVM model, raw spectral data already provided relatively strong classification performance, with accuracies of 90%, 89%, and 88% in the training, cross-validation, and test sets, respectively. After spectral preprocessing, SVM accuracy decreased, except for SD. The SD-SVM model achieved 89% sensitivity, 93% specificity, and 91% accuracy in the test set.

Comparing the three modeling approaches, PLS-DA and SVM exhibited superior performance in the training set relative to KNN, but their predictive accuracy in the test set was slightly lower, suggesting the presence of overfitting. In addition, the influence of spectral preprocessing varied across algorithms. Overall, most models benefited from preprocessing, particularly when SD or SNV methods were applied, highlighting the importance of appropriate spectral treatment in improving classification reliability.

### 3.5. Selection of Characteristic Variables

Selecting informative variables prior to model construction can effectively eliminate redundant spectral information and improve computational efficiency. In this study, three variable selection approaches, including CARS, UVE, and SPA, were employed to identify characteristic wavelengths. For the CARS algorithm, the maximum number of factors and Monte Carlo sampling runs were set to 10 and 100, respectively. During the first 60 sampling iterations, the RMSECV remained relatively stable; however, beyond this point, the error began to increase gradually. Consequently, the 57th sampling run was selected as the optimal iteration.

For the SPA method, effective variables were screened based on RMSE, while the number of latent variables was varied from 1 to 10. In general, the RMSE decreased as the number of variables increased and eventually stabilized. The number of variables corresponding to the minimum RMSE was therefore selected as the optimal subset. When applying UVE, the selection threshold was determined as 99% of the maximum stability of random variables. Spectral variables whose stability values exceeded the upper or lower thresholds were retained as valid inputs for model development.

**Figure 4:**
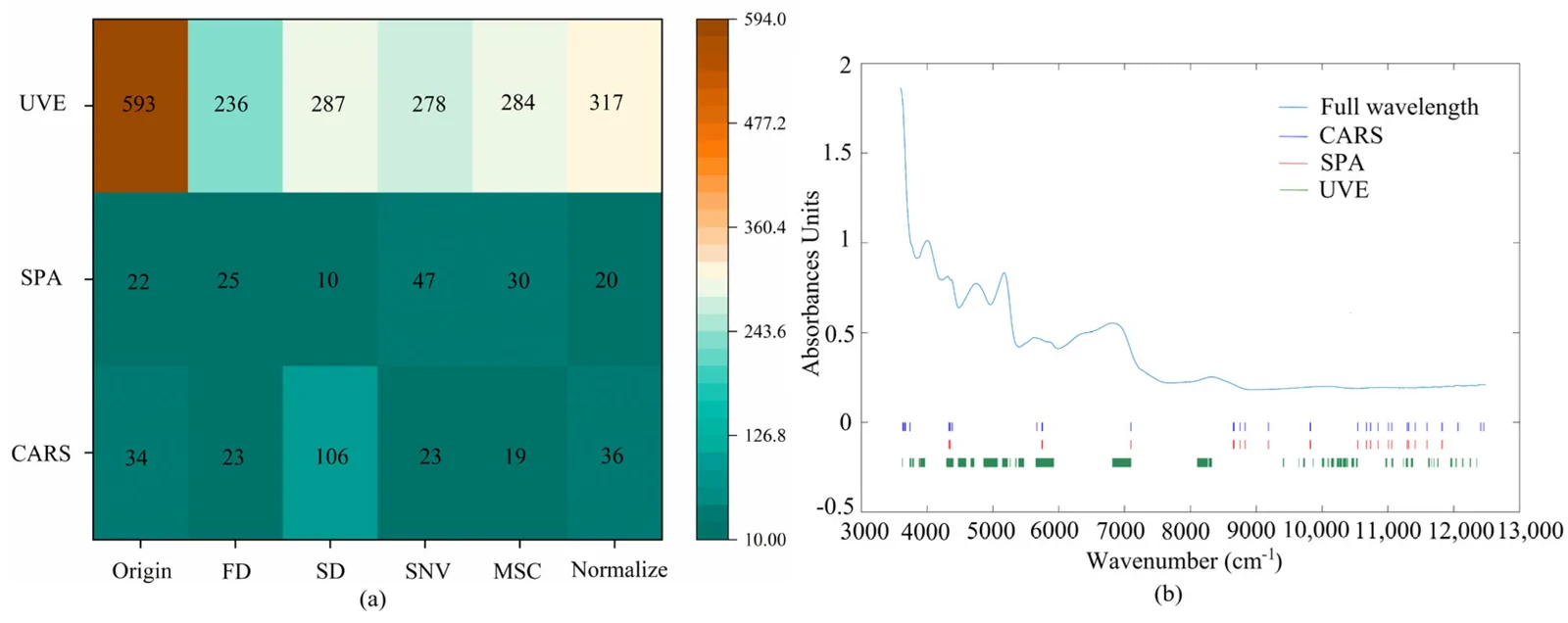
Variable numbers selected by CARS, UVE and SPA based on the different pre-treatments (**a**); The distribution of key variables selected by CARS, UVE and SPA in the original spectrum (**b**).

Figure 4a presents the number of variables selected by CARS, UVE, and SPA under different spectral preprocessing strategies. Among these methods, the Origin-UVE approach selected the largest number of variables (593). In contrast, both CARS and SPA produced substantially smaller subsets. Notably, the SD-SPA method identified only 10 characteristic variables, representing less than 0.86% of the total spectral variables.

Figure 4b illustrates the specific wavelengths selected from the original spectra by different variable selection methods. Although the spatial distributions of the selected variables varied slightly among methods, key absorption regions, particularly around 4381 cm^−1^, 4852 cm^−1^, 6950 cm^−1^, and 8215 cm^−1^, were consistently retained. The peak at 4852 cm^−1^ is attributed to combined-mode O–H stretching and deformation vibrations of bound water and starch. The bands around 6950 cm^−1^ were affected by the combined absorbance of methylene’s C-H, and 8215 cm^−1^ mainly corresponds to C–H second overtone and combination [42,43]. These regions correspond well with the characteristic absorption features identified in the previous spectral analysis. In addition, the variables selected by CARS and SPA were more widely distributed across the spectral range than those obtained by UVE, suggesting that these approaches capture more diverse spectral information while maintaining compact feature sets.

### 3.6. Classification Models Based on Characteristic Variables

After extracting characteristic variables using CARS, SPA, and UVE, classification models based on KNN, SVM, and PLS-DA were constructed. Table 3 summarizes the resulting performances. Overall, the predictive accuracy of PLS-DA models improved after feature selection, whereas the results of KNN and SVM were generally comparable to those obtained using the full spectral range, with the exception of the Origin-UVE-SVM model.

Specifically, after variable selection using UVE, the KNN model exhibited the best predictive performance among the evaluated approaches. The classification accuracies for the training, cross-validation, and test sets reached 94%, 91%, and 92%, respectively, indicating a slight improvement over the corresponding full-spectrum models. For models built with CARS-selected variables, test-set accuracy for KNN, PLS-DA, and SVM was consistently 92%, closely matching performance with the full spectral range. In the CARS-based models, 106, 34, and 23 variables were selected for KNN, PLS-DA, and SVM, respectively.

Among all evaluated approaches, the SD-SPA-PLSDA model showed the best balance between predictive performance and model simplicity. This model employed only 10 variables yet achieved classification accuracies of 92%, 90%, and 92% for the training, cross-validation, and test sets, respectively. Overall, both CARS and SPA maintained or slightly improved the predictive capability of the corresponding full-spectrum models. However, SPA selected substantially fewer variables, thereby reducing computational complexity and simplifying the model structure.

### 3.7. Optimal Model Performance

**Figure 5:**
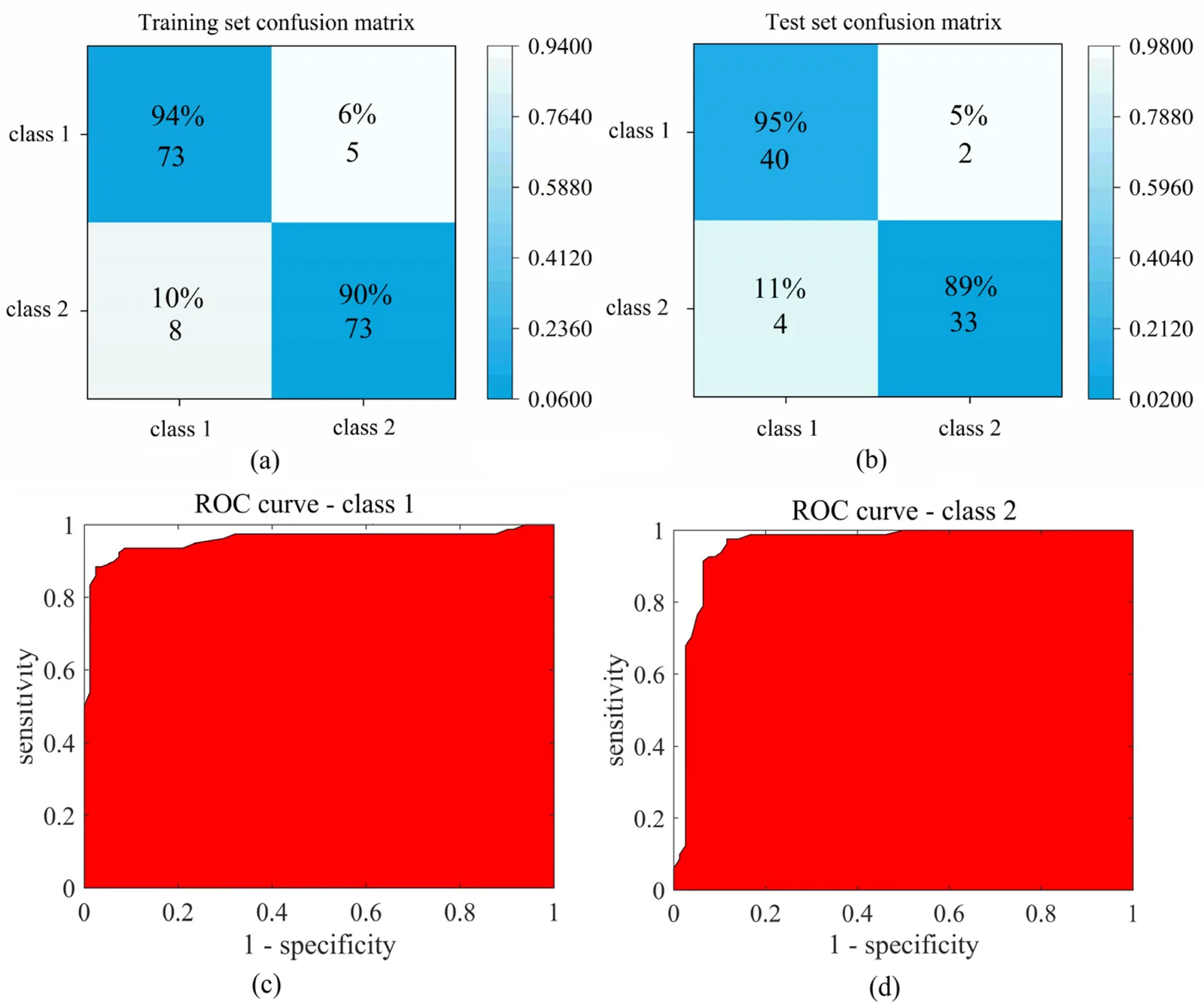
Confusion matrices for the training (**a**) and test sets (**b**), ROC curves of the uncontaminated rice class (**c**) and contaminated rice class (**d**); class 1 is for Cd-contaminated rice and class 2 is for uncontaminated rice.

To further evaluate the performance of the SD-SPA-PLSDA model, confusion matrices for the training and test sets are presented in Figure 5a,b. In the training set, 8 non-contaminated samples (10%) and 5 Cd-contaminated samples (6%) were misclassified. In the test set, the model correctly identified 33 non-contaminated samples (89%) and 40 Cd-contaminated samples (95%), demonstrating reliable classification capability. The ROC curves of the optimal model were shown in Figure 5c,d; the AUCs of both the uncontaminated rice and contaminated rice classes reached 0.96, manifesting satisfactory overall discriminative performance.

### 3.8. Discussion

Cadmium (Cd) contamination in rice poses severe threats to public health and international agricultural trade due to its high bioaccumulation potential and irreversible toxicity to the human renal, skeletal, and cardiovascular systems [4]. Conventional analytical techniques such as ICP-MS provide highly accurate Cd quantification but are time-consuming, labor-intensive, and destructive to samples [9]. In contrast, spectroscopic techniques offer rapid, non-destructive alternatives for contamination screening [12].

The feasibility of rapid discrimination of Cd contamination in rice based on NIR spectroscopy is discussed in this paper. In fact, NIR spectra exhibited negligible visual differences between Cd-contaminated and uncontaminated rice samples. This observation is consistent with previous agricultural spectral studies, as the heavy metal Cd does not absorb energy in the near-infrared region [44]. The subtle spectral variations observed are primarily attributed to Cd-induced structural alterations in rice endogenous biomolecules, including starch, proteins, and lipids. Among the selected characteristic variables in this paper, the band at 4381 cm^−1^ corresponds to the combined C–H and N–H stretching vibrations of starch and protein. The wavenumber at 5669 cm^−1^ is assigned to the second overtone of C–H stretching vibrations associated with rice starch and polysaccharides, while the band at 12,064 cm^−1^ represents the third overtone absorption of C–H stretching vibrations derived from amylose and lipid components [45]. Despite the minimal spectral discrepancies (Figure 2), the developed chemometric models effectively extracted hidden Cd-related spectral features and achieved accurate classification of contaminated and uncontaminated rice samples, validating the feasibility of NIRS coupled with chemometrics for trace Cd contamination screening in rice.

Among the evaluated approaches, PLS-DA demonstrated superior performance on the training set compared with KNN and SVM, whereas the differences among models on the test set were relatively small. Given its strong predictive ability and relatively simple structure, PLS-DA is particularly suitable for NIR-based spectral classification. In recent years, deep learning models such as convolutional neural networks (CNNs) have demonstrated promising performance in spectral analysis. Nevertheless, their practical application in grain detection is constrained by stringent requirements for large-scale sample datasets and high-performance computing resources [46]. By contrast, the classical chemometric methods adopted in this study can achieve reliable classification results with conventional sample sizes. This practical advantage makes the proposed method more accessible and cost-effective for grassroots supervision departments, grain procurement enterprises, and market detection institutions, facilitating its adoption in routine on-site grain safety screening.

In summary, this study demonstrates the great potential of NIRS for rapid, non-destructive screening of Cd-contaminated rice. The optimized PLS-DA model provides an efficient and low-cost preliminary screening strategy that compensates for the low throughput and destructive nature of traditional ICP-MS detection. However, several limitations remain. First, rice samples were collected from limited regions and planting environments; variations in rice cultivars, soil background values, and climatic conditions may limit the model’s universal applicability. Second, this study only achieved qualitative discrimination of Cd contamination without precise quantitative prediction of Cd concentrations. Future work will expand the sample coverage across diverse rice varieties and producing areas, optimize spectral pretreatment and model parameters, and construct high-precision quantitative models.

## 4. Conclusions

This study systematically evaluated spectral preprocessing methods, variable selection strategies, and machine learning classifiers for NIRS-based discrimination of Cd-contaminated rice. SD preprocessing was the most effective spectral treatment for the raw spectra, SPA the optimal variable selection strategy, and PLS-DA the superior classifier. SD preprocessing alone raised KNN and SVM test-set accuracy from 73% and 88% to 93% and 91%, respectively. The SD-SPA-PLS-DA model achieved the best overall performance, with training, cross-validation, and test-set accuracies of 92%, 90%, and 92% using only 10 wavelength variables, effectively eliminating spectral redundancy while preserving discriminative power. These findings establish NIRS coupled with PLS-DA and targeted wavelength selection as a practical, rapid, and accurate platform for Cd-contaminated rice classification, with clear implications for food safety surveillance and precision agriculture.

## Figures and Tables

**Table 1:** Cd contents in rice samples.

Compound	Sample Set	Total Samples	Content (mg/kg)	Mean (mg/kg)	SD	Non-Contaminated Samples	Contaminated Samples
Cd	Training set	159	0.005~1.86	0.39	0.43	81	78
	Test set	79	<LOQ~1.83	0.41	0.44	37	42

Note: LOQ indicates limit of quantification.

**Table 2:** Results for the discrimination of contaminated rice based on spectral preprocessing methods.

Model	Preprocessing Methods	Training Set			Cross-Validation Set			Test Set		
		Sens (%)	Spec (%)	Accu (%)	Sens (%)	Spec (%)	Accu (%)	Sens (%)	Spec (%)	Accu (%)
KNN	Origin	86	65	76	88	68	78	81	64	73
	FD	95	83	89	96	85	91	78	88	83
	SD	95	83	89	95	86	91	92	93	93
	SNV	88	88	88	85	86	86	89	74	82
	MSC	88	88	88	85	86	86	86	79	83
	Normalize	93	79	86	93	81	87	86	90	88
PLSDA	Origin	90	92	91	90	88	89	92	88	90
	FD	100	100	100	90	87	89	86	88	87
	SD	100	100	100	89	91	90	84	93	89
	SNV	89	94	92	90	91	91	92	90	91
	MSC	90	94	92	90	91	91	97	57	77
	Normalize	93	92	93	90	92	91	92	86	89
SVM	Origin	89	91	90	89	90	89	89	86	88
	FD	88	94	91	88	94	91	89	88	89
	SD	94	88	91	90	90	90	89	93	91
	SNV	90	90	90	90	90	90	92	83	88
	MSC	100	100	100	84	81	83	97	45	71
	Normalize	95	88	92	91	90	91	89	79	84

Note: Sens indicates Sensitivity; Spec indicates Specificity; Accu indicates Accuracy.

**Table 3:** Results for the discrimination of contaminated rice based on different algorithm methods and characteristic variables.

Model	Data Type	Pretreat Method	Variables	Training Set			Cross-Validation Set			Test Set		
				Sens (%)	Spec (%)	Accu (%)	Sens (%)	Spec (%)	Accu (%)	Sens (%)	Spec (%)	Accu (%)
KNN	Full	SD	1154	95	83	89	95	86	91	92	93	92
	CARS	SD	106	94	87	91	95	88	92	92	93	92
	SPA	SD	10	88	90	89	88	92	90	89	92	91
	UVE	SNV	278	94	94	94	90	91	91	92	93	92
PLSDA	Full	SNV	1154	89	94	91	90	91	91	92	90	91
	CARS	origin	34	91	94	92	89	92	91	92	93	92
	SPA	SD	10	94	90	92	92	88	90	95	89	92
	UVE	MSC	284	94	94	94	91	90	90	92	93	92
SVM	Full	SD	1154	94	88	91	90	90	90	89	93	91
	CARS	FD	23	88	92	90	85	92	89	92	93	92
	SPA	SD	10	88	94	91	88	94	91	89	92	91
	UVE	Origin	593	89	91	90	89	92	91	89	86	87

Note: Sens indicates sensitivity; Spec indicates specificity; Accu indicates Accuracy.

## Data Availability

The original contributions presented in this study are included in the article/Supplementary Material. Further inquiries can be directed to the corresponding author.

## Supplementary Materials

The following supporting information can be downloaded at: https://www.mdpi.com/article/10.3390/foods15173000/s1, Table S1. Spiked-recovery results for cadmium content in rice.

## References

[1] Yi X., Li C. (2022). Main controllers for improving the resistant starch content in cooked white rice. Food Hydrocoll..

[2] Yan Y., Mao Z., Wang X., Chen Z., Ma C., Wei D., Yan W., Wu X., Guo Y., Xu H. (2025). From farm to table: Assessing the status and health risk assessment of heavy metal pollution in rice in Henan Province. Front. Public Health.

[3] Ochoa M., Tierra W., Tupuna-Yerovi D.S., Guanoluisa D., Otero X.L., Ruales J. (2020). Assessment of cadmium and lead contamination in rice farming soils and rice (*Oryza sativa* L.) from Guayas province in Ecuador. Environ. Pollut..

[4] Charkiewicz A.E., Omeljaniuk W.J., Nowak K., Garley M., Nikliński J. (2023). Cadmium toxicity and health effects—A brief summary. Molecules.

[5] Ishimaru Y., Takahashi R., Bashir K., Shimo H., Senoura T., Sugimoto K., Ono K., Yano M., Ishikawa S., Arao T. (2012). Characterizing the role of rice NRAMP5 in manganese, iron and cadmium transport. Sci. Rep..

[6] Shi Z.Y., Carey M., Meharg C., Williams P.N., Signes-Pastor A.J., Triwardhani E.A., Pandiangan F.I., Campbell K., Elliott C., Marwa E.M. (2020). Rice grain cadmium concentrations in the global supply-chain. Expo. Health.

[7] Gunduz S., Akman S. (2013). Investigation of arsenic and cadmium contents in rice samples in Turkey by electrothermal atomic absorption spectrometry. Food Anal. Methods.

[8] Rosa R., Granja Arakaki D., Melo E., Leite L., Pereira H., Nogueira da Silva K.R., Avellaneda Guimares R., Freitas K.C., Hiane P.A., Bogo D.G. (2025). Determination of selected metals and metalloids in different types of rice by inductively coupled plasma-optical emission spectrometry (ICP-OES). Biol. Trace Elem. Res..

[9] Lan G., Li X., Jia H., Yu X., Wang Z., Yao J., Mao X. (2022). Fast and sensitive determination of cadmium and selenium in rice by direct sampling electrothermal vaporization inductively coupled plasma mass spectrometry. Molecules.

[10] Tang L., Dong J.Y., Qu M.M., Lv Q.M., Zhang L.P., Peng C., Hu Y.Y., Li Y.K., Ji Z.Y., Mao B.G. (2022). Knockout of OsNRAMP5 enhances rice tolerance to cadmium toxicity in response to varying external cadmium concentrations via distinct mechanisms. Sci. Total Environ..

[11] Shimizu H., Ishikawa G., Aoki H., Nakata M., Tanaka J. (2025). Development of a KASP marker set for high-throughput genotyping in Japanese barley breeding programs with various end-use purposes. Breed. Sci..

[12] Cardoso K.L.R., de Jesus J.C., Dargère A.F., da Capela A.P., Oliveira T.D., Carvalho G.R., Santos L.S., Faria P.B., Ferrao S.P.B. (2025). Discrimination of artisanal Minas cheeses according to geographical origin using spectroscopic and chromatographic techniques associated with chemometrics. Food Chem..

[13] Zhu Q., Gao Y.L., Yang B., Zhao K.J., Wang Z.H., Huang F.D., Cheng F.M., Zhao Q., Huang J. (2025). Advanced data-driven interpretable analysis for predicting resistant starch content in rice using NIR spectroscopy. Food Chem..

[14] Wang Q.Y., Li F.S., Jiang X.Y., Hao J., Zhao Y.C., Wu S.L., Cai Y.Y., Huang W.G. (2022). Quantitative analysis of soil cadmium content based on the fusion of XRF and Vis-NIR data. Chemom. Intell. Lab. Syst..

[15] Díaz E.O., Iino H., Koyama K., Kawamura S., Koseki S., Lyu S. (2023). Non-destructive quality classification of rice taste properties based on near-infrared spectroscopy and machine learning algorithms. Food Chem..

[16] Wang L., Wang W., Huang Z., Zhen S., Wang R. (2024). Discrimination of internal crack for rice seeds using near infrared spectroscopy. Spectrochim. Acta A.

[17] Huang F., Peng Y., Li L., Ye S., Hong S. (2023). Near-Infrared spectroscopy combined with machine learning methods for distinguishment of the storage years of rice. Infrared Phys. Technol..

[18] Miao X., Miao Y., Tao S.H., Liu D.B., Chen Z.W., Wang J.M., Huang W.D., Yu Y.Y. (2021). Classification of rice based on storage time by using near infrared spectroscopy and chemometric methods. Microchem. J..

[19] Tong P., Lim J.K., Wei T., Untzizu E., Zhang H., Jiang Y., Cao W. (2021). Rapid identification of the variety and geographical origin of Wuyou No. 4 rice by fourier transform near-infrared spectroscopy coupled with chemometrics. J. Cereal Sci..

[20] Wongsaipun S., Theanjumpol P., Kittiwachana S. (2021). Development of a universal calibration model for quantification of adulteration in Thai jasmine rice using near-infrared spectroscopy. Food Anal. Methods.

[21] Gu Y., Wang P., Zhang S., Dai J., Chen H.P., Lombi E., Howard D.L., van der Ent A., Zhao F.J., Kopittke P.M. (2020). Chemical speciation and distribution of cadmium in rice grain and implications for bioavailability to humans. Environ. Sci. Technol..

[22] Liu Y., Xu L., Zeng S., Qiao F., Jiang W., Xu Z. (2022). Rapid detection of mussels contaminated by heavy metals using near infrared reflectance spectroscopy and a constrained difference extreme learning machine. Spectrochim. Acta A.

[23] Huang Y., Du G., Ma Y., Zhou J. (2021). Predicting heavy metals in dark sun-cured tobacco by near-infrared spectroscopy modeling based on the optimized variable selections. Ind. Crops Prod..

[24] Wang Z., Deng J., Ding Z., Jiang H. (2024). Comparison of optimization algorithms for variable selection to enhance the predictive performance of PLS regression model in determining the concentration of heavy metal Cd in peanut oil. Infrared Phys. Technol..

[25] Gao W.W., Yang Z., Jiang C.J., Sun H.J., Wang Z.W., Zhang M.J., Wei Y., Wang C.T. (2023). Near-infrared reflectance spectroscopy model predictive of cadmium concentration in peanut kernels. J. Food Meas. Charact..

[26] Vega-Castellote M., Pérez-Marín D., Torres-Rodríguez I., Moreno-Rojas J., Ordoñez-Díaz J., Sánchez M. (2023). Green, multivariate approach for obtaining a fingerprint of quality of watermelons at supermarket level using near infrared spectroscopy. LWT-Food Sci. Technol..

[27] Shi X., Gan X.Q., Wang X.B., Peng J.L., Li Z.H., Wu X.Q., Shao Q.S., Zhang A.L. (2022). Rapid detection of Ganoderma lucidum spore powder adulterated with dyed starch by NIR spectroscopy and chemometrics. LWT-Food Sci. Technol..

[28] Rezende-de-Souza J.H., de Moraes-Neto V.F., Pallone J.A.L., Pflanzer S.B. (2025). Recognition of beef aging time using a miniaturized near-infrared spectrometer in tandem with support vector machine. Food Chem..

[29] Qi Z., Wu X., Yang Y., Wu B., Fu H. (2022). Discrimination of the red jujube varieties using a portable NIR spectrometer and fuzzy improved linear discriminant analysis. Foods.

[30] Kwao J.K., Mingle C., Addotey J.N., Opuni K.F.M., Adutwum L.A. (2025). The impact of cluster resolution feature selection on pattern recognition and classification for detecting Sudan dye adulteration in palm oil. Microchem. J..

[31] Li T., Lu C.Y., Huang J.L., Chen Y.Y., Zhang J.X., Wei Y.M., Wang Y.J., Ning J.M. (2023). Qualitative and quantitative analysis of the pile fermentation degree of Pu-erh tea. LWT-Food Sci. Technol..

[32] Lin X.W., Liu R.H., Wang S., Yang J.W., Tao N.P., Wang X.C., Zhou Q., Xu C.H. (2023). Direct identification and quantitation of protein peptide powders based on multi-molecular infrared spectroscopy and multivariate data fusion. J. Agric. Food Chem..

[33] Li H.D., Liang Y.Z., Xu Q.S., Cao D.S. (2009). Key wavelengths screening using competitive adaptive reweighted sampling method for multivariate calibration. Anal. Chim. Acta.

[34] Xu Y., Liu J., Sun Y., Chen S., Miao X. (2023). Fast detection of volatile fatty acids in biogas slurry using NIR spectroscopy combined with feature wavelength selection. Sci. Total Environ..

[35] Brereton R.G. (2022). Numerical introduction to principal components analysis. J. Chemom..

[36] Deng G., Li J., Liu H., Wang Y. (2024). A fast method for predicting adenosine content in porcini mushrooms using Fourier transform near-infrared spectroscopy combined with regression model. LWT-Food Sci. Technol..

[37] (2022). National Food Safety Standard-Limits of Contaminants in Food.

[38] Kennard R.W., Stone L.A. (1969). Computer aided design of experiments. Technometrics.

[39] Peng H., Yi L., Fan X., Zhang J., Gu Y., Wang S. (2025). Near-infrared spectroscopy assisted by random forest for predicting the physicochemical indicators of yak milk powder. Food Chem..

[40] Wei Y., Li X., He Y. (2021). Generalisation of tea moisture content models based on VNIR spectra subjected to fractional differential treatment. Biosyst. Eng..

[41] Shi S., Feng J.H., Yang L.C., Xing J.Y., Pan G.F., Tang J.C., Wang J., Liu J., Cao C.G., Jiang Y. (2023). Combination of NIR spectroscopy and algorithms for rapid differentiation between one-year and two-year stored rice. Spectrochim. Acta A.

[42] Yang H.E., Kim N.W., Lee H.G., Kim M.J., Sang W.G., Yang C.J., Mo C. (2024). Prediction of protein content in paddy rice (*Oryza sativa* L.) combining near-infrared spectroscopy and deep-learning algorithm. Front. Plant Sci..

[43] Bagchi T.B., Sharma S., Chattopadhyay K. (2016). Development of NIRS models to predict protein and amylose content of brown rice and proximate compositions of rice bran. Food Chem..

[44] Liu Y., Xu L., Wang R., Qiao F., Xiong J., Xu Z. (2022). Study on the detection of heavy metal lead (Pb) in mussels based on near-infrared spectroscopy technology and a REELM classifier. Microchem. J..

[45] Chu X.L. (2019). Practical Guide to Near-Infrared Spectral Interpretation.

[46] Li P., Jin Q., Liu H., Han L., Li C., Luo Y. (2024). Determination of soluble solids content in loquat using near-infrared spectroscopy coupled with broad learning system and hybrid wavelength selection strategy. LWT-Food Sci. Technol..

