# Supplementary material for: Identification of Cadmium Contamination in Rice Using Near-Infrared Reflectance Spectroscopy and Machine Learning
Source: Foods. 2026 Aug 26;15(17):3000. doi: 10.3390/foods15173000 (PMC13564494; doi:10.3390/foods15173000)
Supplement: Supplementary file 1 [file foods-15-03000-s001.zip › foods-4504211-supplementary.pdf]

Table S1 Spiked-recovery results for cadmium content in rice (n=3)

Element	Spiking level (mg/kg)	Recovery (%)	RSD (%)
Cd	0.1	96.5	1.2
	0.3	99.3	2.1
	0.5	97.8	1.3
